# Dynamic SOX2 gene expression revealed by phase-separated single RNA imaging in live porcine cells

**DOI:** 10.1016/j.jbc.2026.113424

**Published:** 2026-08-11

**Authors:** Chaoqian Jiang, Zhongyu Yuan, Chuanpei Li, Tianze Li, Jiayan Wu, Dongyan Yang, Xueqing Liu, Zhuoran Yu, Yuchang Yao, Zhonghua Liu, Jun Song, Yanshuang Mu

**Affiliations:** 1 Key Laboratory of Animal Cellular and Genetic Engineering of Heilongjiang Province, Northeast Agricultural University, Harbin, China; 2 College of Life Science, Northeast Agricultural University, Harbin, China; 3 College of Animal Science and Technology, Northeast Agricultural University, Harbin, China

**Keywords:** pig, MS2/MCP system, RNA image, foldon-MS2/MCP system

## Abstract

RNA plays a central role in the formation of diverse traits in pigs. Visualizing RNA dynamics at the single-molecule level in living cells is therefore essential for understanding gene-expression regulation in this species. We report an optimized MS2-MCP RNA-labeling system that incorporates a foldon trimerization domain fused to a fluorescent protein and MCP, thereby enhancing fluorescence signals through liquid-liquid phase separation. Using this Foldon-MS2/MCP system, we successfully imaged endogenous *GFP* mRNA foci in both the nucleus and cytoplasm of living pig cells. Compared with the conventional MS2-MCP system, the Foldon-MS2/MCP system showed markedly increased fluorescence intensity and an improved fluorescence signal-to-noise ratio. We further applied this approach to the endogenous porcine *SOX2* gene, achieving high-resolution tracking of *SOX2* mRNA foci in live pig cells. Moreover, the system enabled real-time visualization of *SOX2* mRNA elongation and translation dynamics. The Foldon-MS2/MCP system provides a powerful platform for investigating RNA localization, gene-expression activation, and endogenous mRNA dynamics in living pig cells.

Pigs are important agricultural livestock and widely used model organisms (1). Elucidating RNA-level molecular mechanisms is critical for understanding gene regulation and cellular organization in pigs (2). RNAs are highly dynamic molecules that not only transmit genetic information from DNA to protein but also function independently in diverse regulatory and structural roles (3, 4, 5). Their life cycle—including transcription, processing, nuclear export, localization, translation, and degradation—requires precise spatiotemporal regulation (6, 7, 8). Consequently, visualizing RNA molecules at the single-molecule level in living pig cells represents a powerful approach for dissecting RNA function (9, 10).

Several strategies have been developed for live-cell RNA imaging, including RNA-binding protein-fluorescent protein fusion approaches (11, 12), CRISPR-based labeling system (3, 13), and fluorophore-RNA aptamer systems (7, 14, 15). Among these, the MS2-MCP system has become the standard method for single-molecule RNA tracking. In this approach, short RNA stem-loop MS2-binding sites (MBSs) are inserted into target transcripts, typically within the 3′or 5′ UTR (16). Fluorescently tagged MS2 coat proteins (MCPs) bind to the MBSs, generating discrete fluorescent foci that correspond to individual RNA molecules and enabling real-time observation of transcription, transport, localization, and translation in living cells (9, 17, 18, 19, 20, 21).

Despite its broad utility, MS2-MCP-based imaging of endogenous RNAs at single-molecule resolution in live pig cells remains uncommon (22). A major limitation is the low abundance of many endogenous transcripts, which limits the number of fluorescently bound MCPs and yields weak signals (18, 23). In addition, unbound MCP-fluorescent protein fusions contribute substantial background fluorescence, thereby reducing the signal-to-noise ratio (SNR) (6, 18). To overcome these challenges, strategies such as split superfolder fluorescent proteins have been developed; these proteins are divided into nonfluorescent fragments that reassemble and emit fluorescence only upon binding to target mRNAs, thereby reducing background signals. In the CRISPR-mediated fluorescence *in situ* hybridization amplifier system (CRISPR FISHer), dCas9 is combined with PP7-aptamer-modified guide RNAs to recruit PP7 coat protein, GFP, and a T4 fibronectin trimer, forming a Foldon-GFP-PCP fusion protein (24). This design enables robust visualization of nonrepetitive genomic DNA sequences guided by a single sgRNA by locally enriching fluorescence signals and improving brightness and the signal-to-background (S/B) ratio. Notably, the CRISPR FISHer system enhances the detection of labeled genomic DNA signals *via* liquid-liquid phase separation, suggesting a promising strategy for real-time RNA labeling (24).

In this study, we established a Foldon-MS2/MCP system for RNA imaging in live pig cells. This system enables real-time tracking of both exogenous *GFP* mRNAs and endogenous porcine *SOX2* transcripts with a higher SNR than the conventional MS2/MCP system. We further demonstrate that foldon-mediated liquid–liquid phase separation underlies the enhanced performance of MS2/MCP labeling. Together, our results show that the Foldon-MS2/MCP system supports high-resolution visualization of *SOX2* mRNA dynamics and single-RNA translation kinetics in living pig cells, providing a robust and sensitive platform for single-molecule RNA imaging in this species.

## Results

### Construction of a foldon-MS2/MCP RNA labeling system in living cells

**Figure 1:**
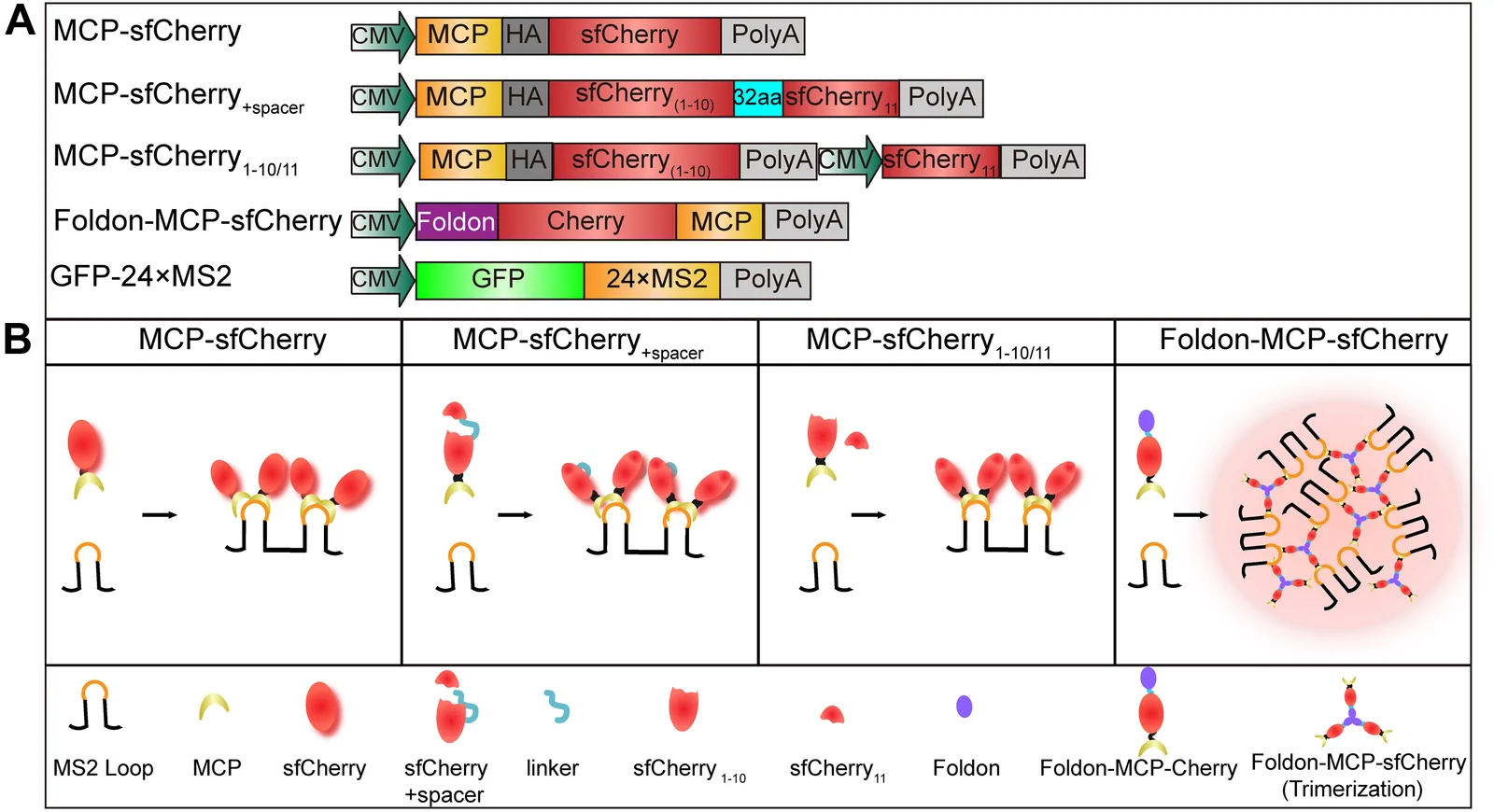
**Construction of MS2/MCP-sfCherry system, MS2/MCP-sfCherry_+spacer_ system, MS2/MCP-sfCherry_1-10/11_ system, MS2/Foldon-sfCherry-MCP system.***A*, schematic of the MCP-sfCherry vector,MCP-sfCherry_+spacer_ vector, MCP-sfCherry_1-10/11_ vector, Foldon-sfCherry-MCP vector. *B*, schematics of fluorescent imaging structural of MCP-sfCherry vector,MCP-sfCherry_+spacer_ vector, MCP-sfCherry_1-10/11_ vector, Foldon-sfCherry-MCP vector.

In the MS2/MCP RNA visualization system, MS2 stem-loop sequences are inserted into target RNAs to recruit RNA-binding motif-fused fluorescent proteins for locus-specific imaging. To enhance labeling efficiency, we fused the foldon domain to the N-terminus of sfCherry and subsequently to the MS2 coat protein (MCP), generating the Foldon-sfCherry-MCP construct (Fig. 1*A*).The foldon domain is a trimerization motif derived from bacteriophage T4 fibritin that promotes stable trimer formation through its C-terminal region. Accordingly, Foldon-sfCherry-MCP is expected to form stabilized trimers, enabling MS2 loop-containing RNAs to recruit trimeric fusion proteins that assemble into phase-separated structures. The proposed assembly models are illustrated in (Fig. 1*B*). Through this mechanism, Foldon-sfCherry-MCP is anticipated to increase local sfCherry accumulation at RNA-targeting loci in live cells, thereby enhancing RNA signal intensity.

**Figure 2:**
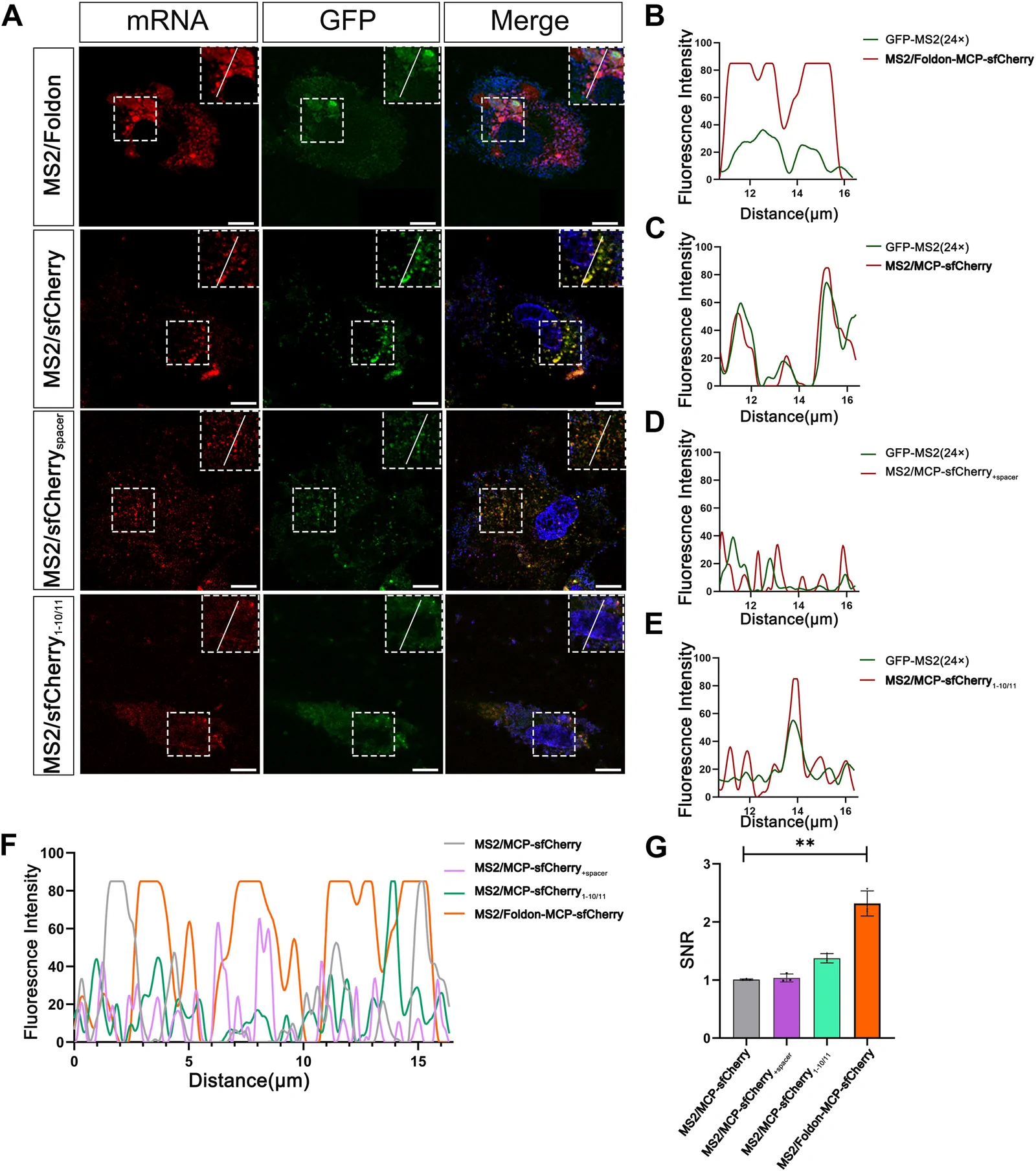
**Labeling of exogenous *GFP* mRNA using MS2/MCP-sfCherry, MS2/MCP-sfCherry_+spacer_, MS2/MCP-sfCherry_1-10/11_, and MS2/foldon-sfCherry-MCP (Foldon-MS2/MCP) in living cells.***A*, Colocalization of *GFP* mRNA (red), labeled by the indicated MCP fusion proteins, with GFP protein (*green*). Nuclei were counterstained with Hoechst 33,342 (*blue*). Insets show magnified merged views of the boxed regions. *B–E,* fluorescence intensity profiles along the white lines in the insets of (*A*), illustrating colocalization of GFP protein and *GFP* mRNA signals across the four constructs. *F*, line plots of *GFP* mRNA fluorescence intensity labeled by each MS2/MCP-sfCherry variant. *G*, quantification of *GFP* mRNA fluorescence signal-to-noise ratio (SNR) for each construct, showing the highest SNR for Foldon-sfCherry-MCP(n ≥ 3, *p* < 0.01).

To evaluate labeling performance, we compared four MS2/MCP systems using different MCP fusion proteins: MCP-sfCherry, MCP-sfCherry with a spacer, MCP-sfCherry_1-10/11_, and Foldon-sfCherry-MCP (Fig 1). Porcine embryonic fibroblast (PEF) cells were transiently transfected with EGFP-24 × MS2 together with each MCP-fused fluorescent construct for 48 h. Confocal microscopy revealed distinct fluorescent puncta in all groups, with GFP protein signals colocalizing with *GFP* mRNA labeled *via* the MS2/MCP system (Fig. 2, *A*–*E*). Line-scan intensity profiles confirmed signal colocalization, and quantitative analyses demonstrated that the Foldon-sfCherry-MCP group exhibited the highest fluorescence intensity and signal-to-noise ratio (SNR) (Fig. 2, *F* and *G*), indicating superior labeling efficiency.

**Figure 3:**
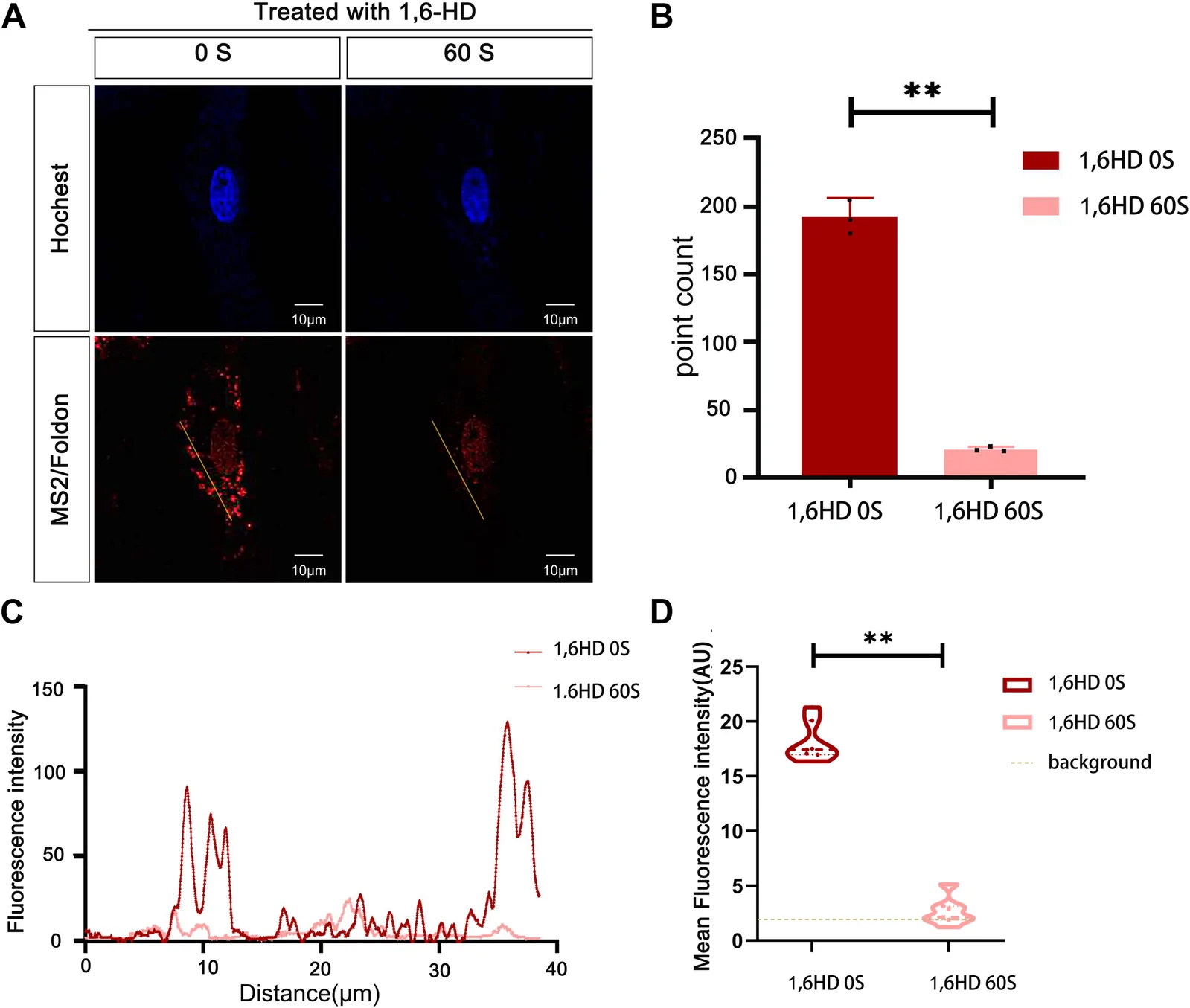
**Disruption of Foldon-sfCherry-MCP condensates by 1,6-hexanediol in living cells.***A*, representative fluorescence images of cells co-expressing GFP-24 × MS2 and Foldon-sfCherry-MCP(MS2/Foldon) before and after 1,6-hexanediol treatment. Scale bars, 10 μm. *B*, quantification of the number of cells displaying detectable GFP mRNA signals in the presence or absence of 1,6-hexanediol (n ≥ 3, *p* < 0.01). *C*, quantification of fluorescence intensity fluctuations within Foldon-sfCherry-MCP condensates in untreated and treated cells. *D*, quantitative detection of GFP mRNA fluorescence intensity in single cells with or without 1,6-hexanediol treatment (n ≥ 3, *p* < 0.01).

To further validate the involvement of foldon-domain-mediated phase separation, cells were treated with 1,6-hexanediol (HD), a reagent known to disrupt weak hydrophobic interactions that underlie phase-separated condensates. Following 60 s of HD treatment, red fluorescent aggregates were markedly reduced, accompanied by signal diffusion and a decrease in fluorescence intensity (Fig. 3*A*). Consistently, *GFP* mRNA signal intensity, aggregation behavior, and the number of labeled cells were significantly reduced after HD treatment (Fig. 3, *B*–*D*), supporting a phase-separation-dependent mechanism for signal enhancement in this system.

### Real-time tracking of exogenous GFP mRNA in living cells

**Figure 4:**
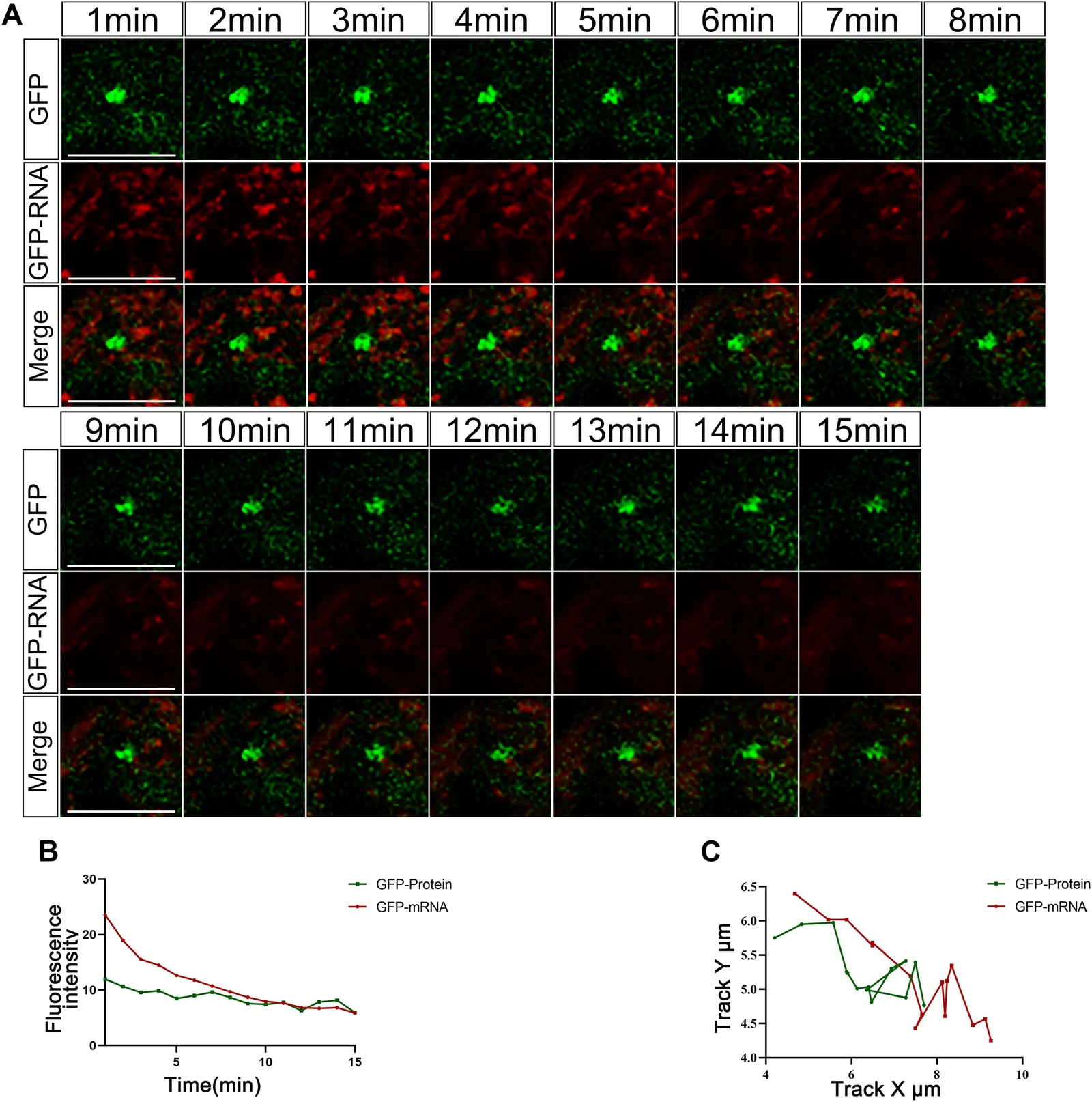
**Dynamic tracking of exogenous *GFP* mRNA using the foldon-sfCherry-MCP system.***A*, time-lapse confocal images showing GFP protein (green) and *GFP* mRNA (red) dynamics in PEF cells over 15 min, acquired at 1-min intervals. Scale bars, 10 μm. *B*, fluorescence intensity traces of GFP protein and *GFP* mRNA corresponding to the time-lapse images in (*A*), llustrating dynamic fluctuations over time. *C*, representative trajectories of GFP protein and *GFP* mRNA movement over 15 min, demonstrating coordinated spatiotemporal dynamics.

To determine whether the Foldon-MS2/MCP system supports dynamic mRNA tracking, we performed 15-min time-lapse imaging at 1-min intervals in cells co-transfected with GFP-24 × MS2 and foldon-sfCherry-MCP (Fig. 4*A*). This approach enabled real-time monitoring of *GFP* mRNA expression and intracellular movement within single living cells (Fig. 4, *B* and *C*).

Live imaging revealed that large GFP-positive foci were predominantly localized to the basal cytoplasm. These foci corresponded to sites of both mRNA accumulation and translation, as GFP protein signals consistently colocalized with the mRNA foci. Together, these observations demonstrate that the Foldon-MS2/MCP system enables efficient, dynamic, and sustained labeling of exogenous *GFP* mRNA in living cells.

### Establishment of a real-time tracking system for endogenous *SOX2* RNA and protein

**Figure 5:**
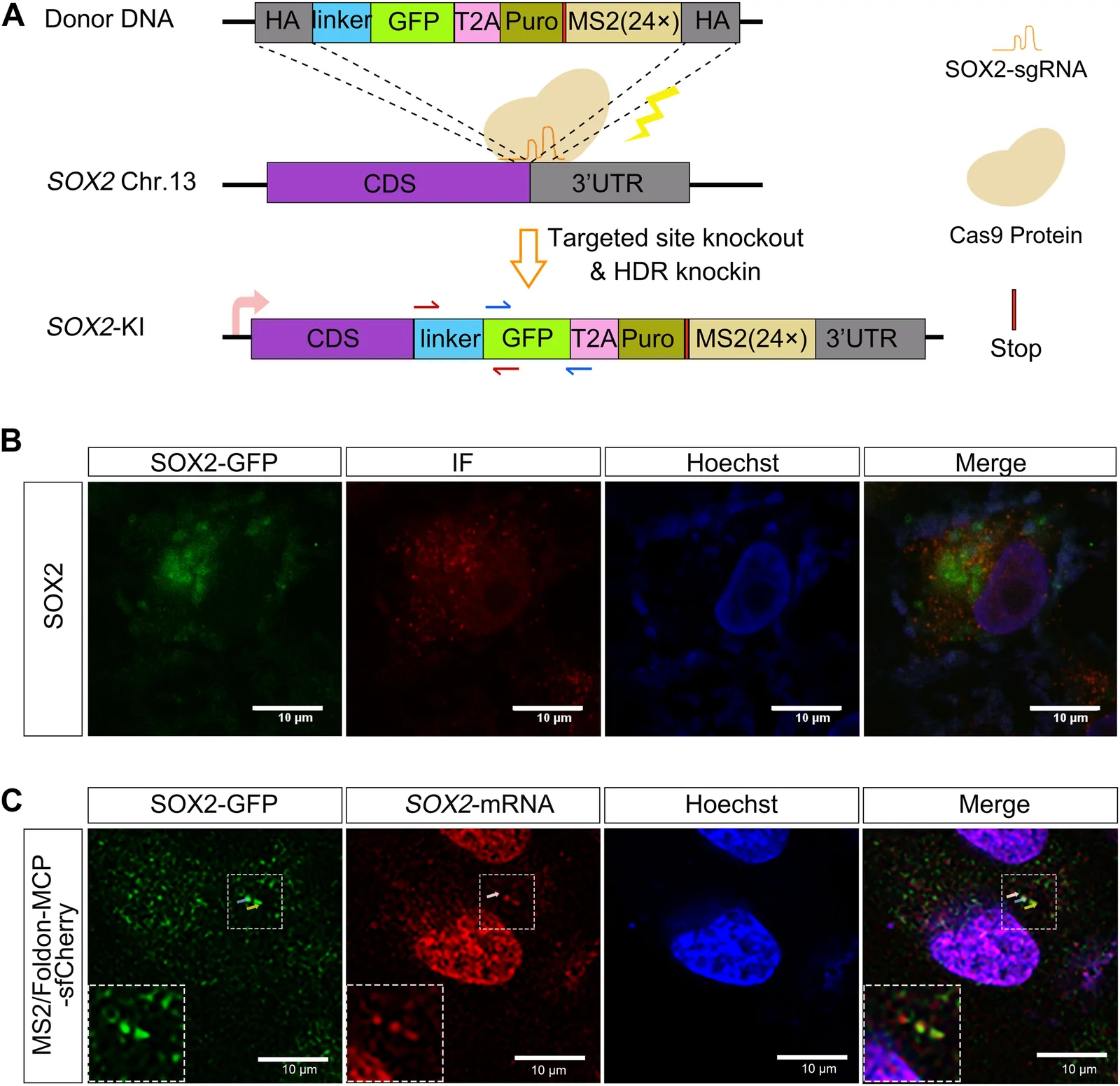
**Establishment of an endogenous SOX2 reporter system by knock-in of GFP and 24 × MS2 loops.***A*, schematic of the CRISPR/Cas9-mediated knock-in strategy targeting the 3′ end of the *SOX2* coding region. The donor construct includes a left homology arm (HA), eGFP coding sequence, puromycin resistance gene (Puro) and the endogenous stop codon (TGA), 24 × MS2 stem-loop cassette, and a right homology arm (HA), resulting in expression of a SOX2-GFP fusion protein and *SOX2* mRNA tagged with 24 × MS2 loops. *B*, immunofluorescence analysis of *SOX2* expression in SOX2-GFP knock-in PK15 cells. Nuclei were counterstained with Hoechst 33,342 (*blue*). *C*, representative images showing colocalization of SOX2-GFP protein (*green*) and *SOX2*-24 × MS2 mRNA labeled by Foldon-sfCherry-MCP (red) in living PK15 cells. Nuclei were counterstained with Hoechst 33,342 (*blue*).

To extend the Foldon-MS2/MCP system to endogenous transcripts, we targeted the porcine *SOX2* gene. A CRISPR/Cas9-mediated knock-in strategy was used to insert a GFP-T2A-Puro-TGA-24 × MS2 cassette into the 3′ UTR of *SOX2* (Fig. 5*A*). Single-guide RNAs targeting the 3′ UTR were pre-screened for high editing efficiency, and stable knock-in cell lines were established by puromycin selection.

Correct integration was confirmed by GFP expression and immunofluorescence detection of SOX2 protein (Fig. 5*B*). Upon transfection of foldon-sfCherry-MCP into these cells, confocal imaging revealed robust red-green fluorescence colocalization in the cytoplasm (Fig. 5*C*), validating the simultaneous visualization of endogenous SOX2 protein and *SOX2* mRNA in living cells.

### Dynamic imaging of *SOX2* mRNA movement and translation kinetics

**Figure 6:**
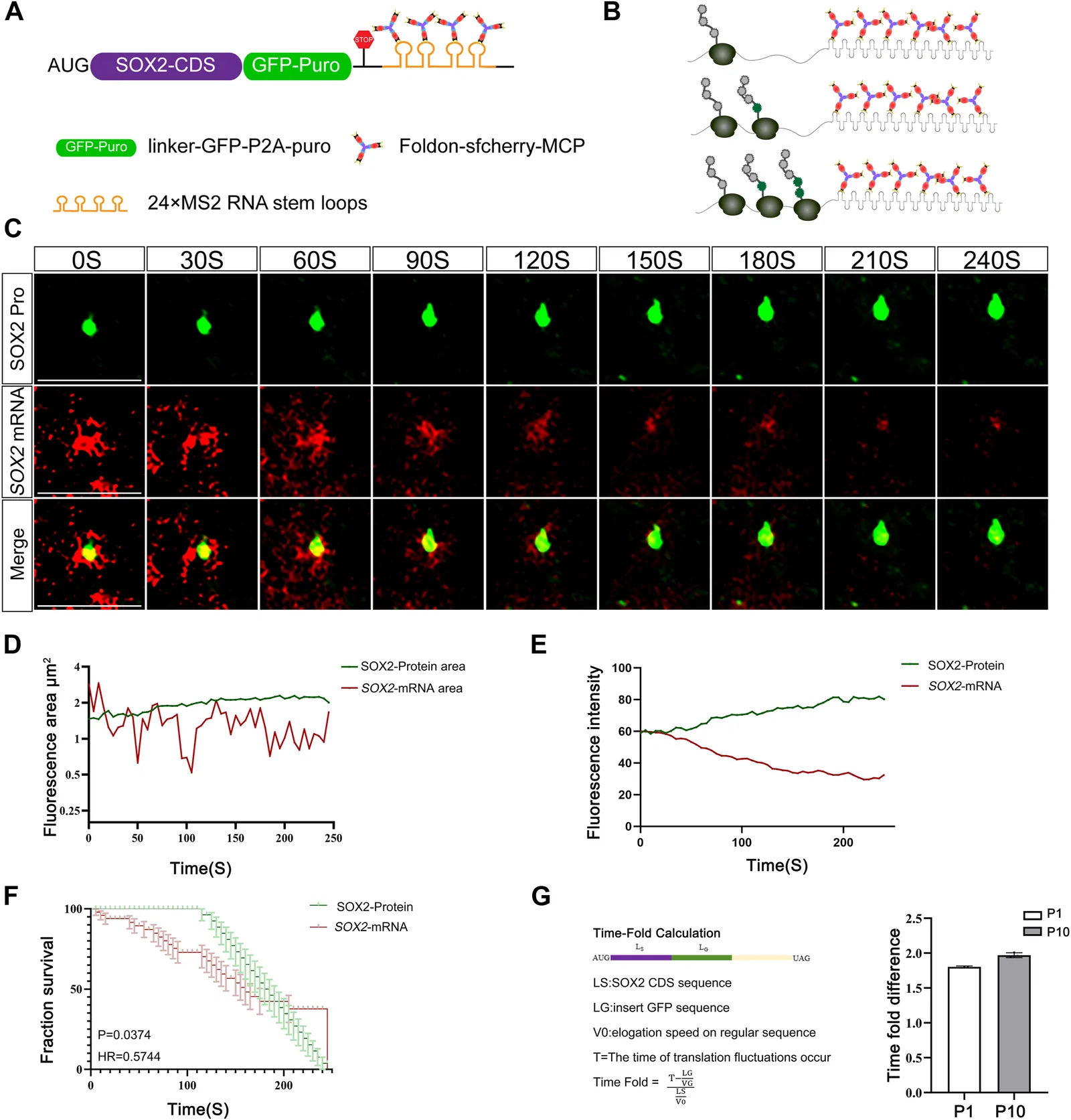
**Dynamic Imaging of SOX2 mRNA Movement and Translation Kinetics Using the Foldon-MS2/MCP system.***A*, schematic of the construct designed for single-molecule imaging of *SOX2* mRNA translation, comprising GFP and puromycin resistance (Puro) coding sequences upstream of the stop codon, followed by 24 × MS2 stem-loop sequences inserted into the 3′UTR1 region. MS2-binding sites (MBSs) are recognized by foldon-sfCherry-MCP to enhance signal intensity. *B*, sSchematic of the ribosome runoff assay used to estimate the translational elongation speed of *SOX2* mRNA. *C*, time-lapse fluorescence images showing dynamic movement of SOX2 protein and mRNA in PK15 cells labeled with the Foldon-MS2/MCP system. Images were acquired every 240 s. Scale bars, 10 μm. *D*, quantification of cytoplasmic fluorescence area for SOX2 protein (*green*, SOX2-GFP) and *SOX2* mRNA (red, Foldon-sfCherry-MCP) based on single-molecule tracking. *E*, temporal changes in cytoplasmic fluorescence intensity of SOX2 protein (*green*) and *SOX2* mRNA (*red*), revealing dynamic endogenous expression patterns. *F*, survival curves comparing the stability of SOX2 protein (*green*) and *SOX2* mRNA (*red*) over time, showing greater persistence of the protein signal. *G*, estimation of translational elongation speed based on the median ribosome runoff time.

Time-lapse imaging of *SOX2* mRNA in PK15 cells was performed at 4-min intervals using the Foldon-MS2/MCP system. The experimental principle and imaging workflow are illustrated in Figure 6, *A* and *B*. Confocal microscopy revealed pronounced spatiotemporal dynamics of labeled *SOX2* mRNA in living cells (Fig. 6*C*).

Fluorescence trajectories of SOX2-linker-EGFP were extracted using the TrackMate plugin in ImageJ, enabling quantitative analysis of mRNA and protein movements within individual cells (Fig. 6, *D*–*E*). Survival curve analysis further demonstrated that *SOX2* mRNA signals decayed more rapidly than the corresponding protein signals (Fig. 6*F*). Survival curve analysis revealed distinct persistence profiles between SOX2 mRNA and the corresponding protein signals (Fig. 6*F*). In contrast, SOX2 protein signals were more stable and persisted longer, with statistical significance supported by *p*-value and hazard ratio analyses.

To assess system robustness over extended culture, translation rates were compared between early-generation and late-generation labeled cells (Fig. 6*G*). Comparable translation kinetics were observed across passages, indicating stable labeling performance and sustained translational activity over long-term cell propagation.

### High-resolution tracking of SOX2 protein translation dynamics

**Figure 7:**
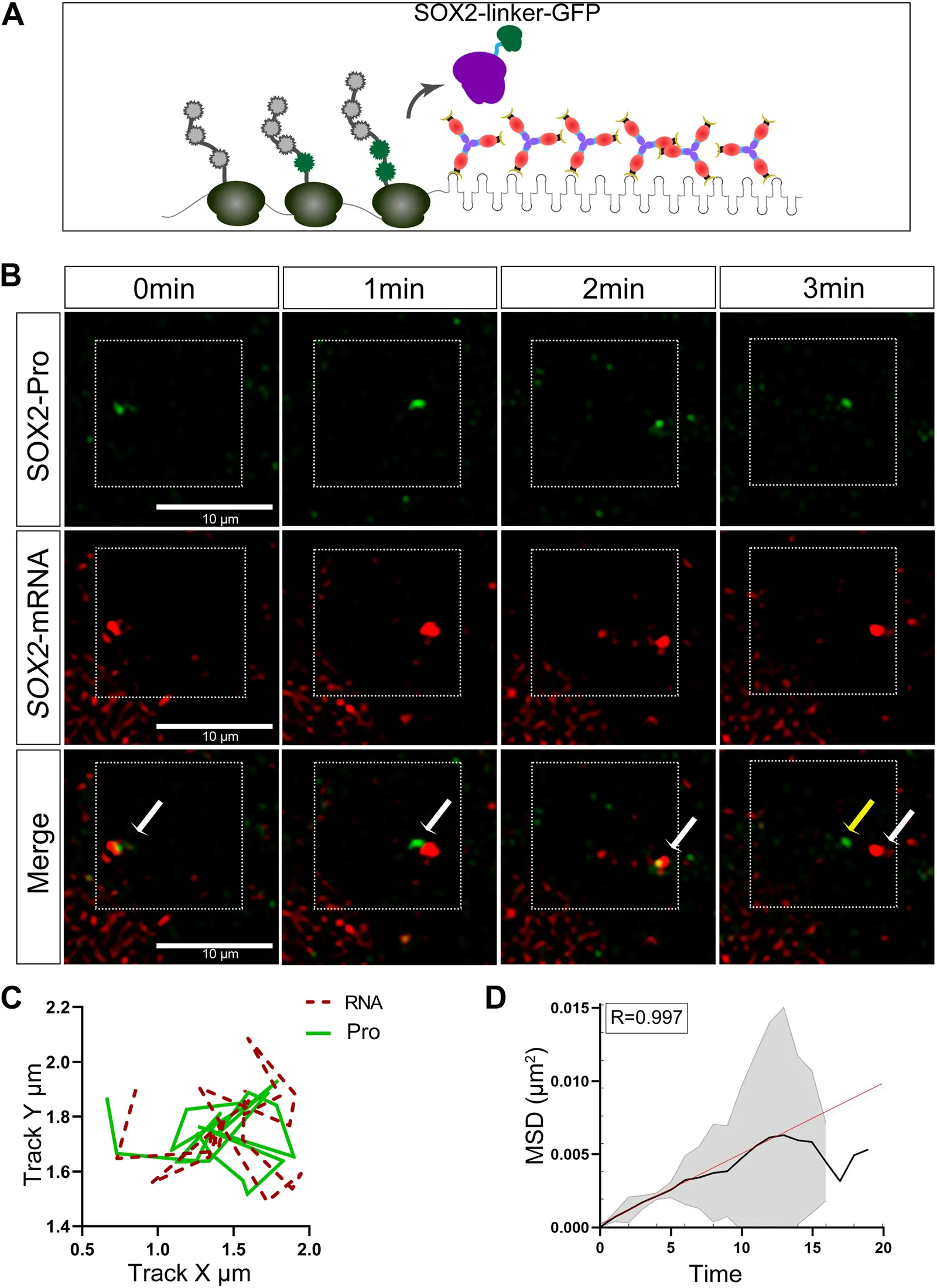
**Dynamic tracking of endogenous SOX2 mRNA using the Foldon-MS2/MCP system system.***A*, schematic illustration of fluorescence recovery after photobleaching (FRAP), in which fluorescence reflects ongoing ribosomal synthesis of EGFP, while newly arriving ribosomes generate nascent polypeptides (NAPs, *green*). *B*, live-cell snapshot showing co-localization and co-expression of SOX2-GFP-24 × MS2 with Foldon-sfCherry-MCP. Green: SOX2-linker-GFP; *red*: foldon-sfCherry-MCP. Scale bar: 10 μm. Visualization of translation dynamics of single mRNAs in live cells. *White arrows*: colocalization; *yellow arrows*: separation. *C*, coordinated movement of *SOX2* mRNA (*red*) and the translation sites (translation sites, *green*) over time.Arrows and squares indicate tracked fluorescent points, illustrating single-molecule translation dynamics in living cells. *D*, mean square displacement (MSD) analysis of *SOX2* mRNA shown in (C).MSD analysis of SOX2 mRNA trajectories. The *black line* represents experimental MSD values, and the red line indicates linear fitting. Shaded area represents standard deviation. The model showed a strong fit (R = 0.997).

To resolve translation dynamics at higher temporal resolution, SOX2-GFP protein was tracked in single cells over a 3-min interval (Fig. 7). At the early stages of translation, the *translation sites* and the mRNA labeling site exhibited coordinated movement. Upon completion of translation, the red mRNA signal and green protein signal are separated, reflecting dissociation of the nascent polypeptide from the transcript.

Detailed trajectory mapping confirmed the initial co-migration and subsequent divergence of mRNA and protein signals. Mean square displacement (MSD) analysis was performed to quantify the mobility of *SOX2* mRNA and protein during the transcription-translation process (Fig. 7*C*). MSD analysis suggested constrained diffusion behavior of *SOX2* mRNA (Fig. 7*D*), and the fitted model exhibited a high goodness-of-fit (R = 0.997), consistent with strong spatial anchoring during translation.

## Discussion

In this study, we developed a phase separation-enhanced MS2/MCP RNA labeling system, termed Foldon-sfCherry-MCP, that markedly improves labeling sensitivity and signal-to-noise ratio (SNR) in living pig cells. This system enables robust real-time imaging of single mRNA molecules and permits precise tracking of translation dynamics with high spatiotemporal resolution.

In the Foldon-MS2/MCP system, the foldon domain, a trimerization motif derived from bacteriophage T4 fibritin, drives protein trimerization and multivalent interactions (17, 25, 26). When fused to sfCherry and MCP, the resulting construct binds MS2 stem-loop sequences on target RNAs and assembles into liquid-liquid phase-separated condensates (24, 27). This design generates bright and dynamic fluorescent assemblies at MS2-tagged RNA loci. Direct comparisons with the conventional MS2-MCP system demonstrated substantial gains in fluorescence intensity and signal-to-background ratio (28). Importantly, RNAs labeled with Foldon-sfCherry-MCP retained normal biological behavior, including intracellular transport and active translation, indicating that the enhanced labeling does not disrupt physiological RNA functions (29).In particular, insertion into the 3′UTR avoids disruption of the coding sequence and 5′ UTR regulatory elements, thereby preserving SOX2 translational fidelity and protein localization. The 24 × repeat design further enhances signal-to-noise ratio without substantially affecting mRNA behavior, making it a well-established strategy for single-molecule RNA tracking in living cells (15).

In mammalian cells, mRNAs typically persist for hours to days and undergo tightly regulated, dynamic transitions throughout their life cycle (30). Although existing single-molecule imaging approaches based on bacteriophage stem loops and fluorescent protein-tagged coat proteins have greatly advanced live-cell RNA visualization, maintaining sufficient signal strength during long-term imaging remains challenging (21). Here, the Foldon-MS2/MCP system supported long-term visualization of mRNA dynamics for up to 10 days. Nonetheless, continuous tracking of individual transcripts is still constrained by their movement in and out of the focal plane. Combining this system with emerging high-speed three-dimensional imaging technologies may enable comprehensive tracking of mRNAs and polysomes throughout the entire cellular volume, providing deeper insights into mRNA life cycle regulation (31).

During its lifetime, an mRNA molecule progresses through transcription, processing, nuclear export, cytoplasmic transport, storage, translation, and degradation (32). How these processes are coordinated, particularly the coupling of translation with other cytoplasmic events, remains incompletely understood. The imaging strategy described here provides a direct, real-time readout of translation activity, capturing transient regulatory events with high spatial and temporal precision (33). Using this approach, we successfully visualized both exogenous *GFP* mRNA and endogenous SOX2 transcripts in living porcine cells (34). Notably, the system preserves accurate spatial information on translation sites, enabling analysis of polysome dynamics and localized translation within defined subcellular regions (35).The observed colocalization between GFP and mRNA signals does not necessarily represent active translation sites but may arise from nascent protein proximity, transient SOX2 cytoplasmic localization, or imaging resolution limits, and should therefore be interpreted with caution (36).

Real-time imaging further revealed coordinated dynamics between mRNAs and their translated proteins, with proteins exhibiting longer persistence than their corresponding mRNAs, likely reflecting differences in degradation kinetics. Mean-square displacement analysis indicated restricted cytoplasmic diffusion of *SOX2* mRNA, consistent with its localization to specific subcellular compartments or with stable association with the translation machinery. SOX2-GFP showed predominant nuclear localization, consistent with its function as a transcription factor, At the same time, while SOX2 mRNA was detected in both nuclear and cytoplasmic compartments, suggesting spatially regulated mRNA distribution and translation before nuclear import. This compartmentalized expression pattern suggests that localized translation may fine-tune SOX2 protein levels, enabling rapid buffering of transcriptional fluctuations and dynamic adjustment of pluripotency-associated gene regulatory networks in response to microenvironmental cues, thereby supporting stem cell self-renewal and lineage priming (7, 37, 38).The phase separation-mediated signal amplification conferred by Foldon-sfCherry-MCP concentrates fluorescent proteins at RNA loci, substantially enhancing detection sensitivity. This feature is particularly advantageous for interrogating translation regulation of low-abundance transcripts at the single-molecule level.

In summary, we established a foldon-enhanced MS2/MCP RNA imaging platform that amplifies labeling signals *via* phase separation, enabling sensitive and high-resolution visualization of both exogenous and endogenous RNAs in living pig cells. Using endogenous *SOX2* mRNA as a model, we demonstrate that this system provides superior fluorescence intensity, improved SNR, and more robust dynamic tracking than the conventional MS2/MCP approach. This platform offers a powerful and versatile tool for studying transcription, RNA transport, translation, and RNA–protein interactions in living animal cells.

## Experimental procedures

### Plasmid vector construction

To construct the pSL-24 × MS2 vector, the 12 × MS2 fragment was amplified from the pSL-12 × MS2 plasmid (Addgene #191123) and inserted into the *Bam*HI site of the linearized pSL-12 × MS2 backbone.

To construct the EGFP-24 × MS2, the EGFP fragment was amplified from the pEGFP-C1 plasmid and cloned into pSL-24 × MS2 using BamHI sites (NEB, Cat.No.R3136V, NY, USA) and *Bgl*II(NEB, Cat.No.R0144V) restriction sites.

To construct MCP-sfCherry, the MCP sequence was amplified from the Cas9(C)-VP64_T2A_MCP-P65-HSF1 vector (Addgene, Vector,Cat. No.101113). An HA tag was introduced *via* PCR, and the MCP fragment was seamlessly assembled with a psfCherry backbone using the NEBuilder HiFi DNA Assembly Kit (NEB, Cat. No. E2621S).

To generate MCP-sfCherry_1-10/11_, the sfCherry sequence was split into two segments *via* PCR, and the CMV promoter was inserted between them using NEBuilder HiFi DNA Assembly Kit (NEB, Cat. No.E2621S).

To construct MCP-sfCherry_+spacer_, a synthetic 32-amino-acid spacer was inserted into the sfCherry sequence of MCP-sfCherry using the same cloning method.

The Foldon-sfCherry-MCP plasmid was created by fusing foldon (a synthetic trimerization domain), sfCherry, and MCP sequences through homologous recombination. The foldon-linker was synthesized as a short oligonucleotide, and the pcDNA3.1 vector backbone was PCR-amplified with homology arms for seamless assembly using the NEBuilder HiFi DNA Assembly Kit (NEB, Cat.No.E2621S).

The pSLEP-24 × MS2 vector (p5′HA-linker-EGFP-T2A-Puro-TGA-24 × MS2-3′HA) was designed to target the porcine SOX2 3′ UTR. It contains a 200 bp 5′ homology arm, EGFP reporter, T2A-puromycin resistance cassette, 24 × MS2 repeats, and a 200 bp 3′ homology arm downstream of the SOX2 stop codon. Homology arms were amplified from porcine genomic DNA(Supplementary Material: Table S1).Linker-EGFP and T2A-Puro fragments were amplified from pEGFP-C1 and lentiCRISPRv2, respectively, and the entire construct was assembled using the NEBuilder HiFi DNA Assembly Kit (NEB, Cat.No.E2621S).

### Cell culture

Primary pig fetal fibroblasts (PFFs) were isolated from 33-day-old Large White pig embryos. All animal procedures were approved by the Institutional Animal Care and Use Committee (IACUC) of Northeast Agricultural University. PFFs were cultured in DMEM(Gibco,Cat. No.12491015) supplemented with 15% FBS(FBS;Gibco,Cat. No.A5256701), 1% penicillin-streptomycin (CELLGRO,Cat. No. 30-002-CI), 1% nonessential amino acids (Invitrogen, Cat. No. 11140-050), and 2 mM L-glutamine(Sigma,Cat. No. G8540-100G) at 37 °C with 5% CO_2_.

Porcine kidney epithelial cells (PK15) were cultured in DMEM(Gibco,Cat. No.12491015) with 15% FBS(FBS;Gibco,Cat. No.A5256701). Cells were passaged using 0.25% trypsin-EDTA(Thermo, Cat.No.15400054) and seeded into six- or 12-well plates upon reaching 80% confluence.

### Cell transfection and selection

Approximately 1 × 10^6^ PK15 cells were trypsinized, pelleted, and resuspended in 20 μl Opti-MEM(Gibco,Cat. No. 31985-070) with 5 μg plasmid DNA. Electroporation was performed using the CUY21 electroporator (BEX, CUY21 EDIT II). After 48 h, cells were subjected to puromycin (1 mg/ml) (Sigma,Cat.L2630-25MG) selection for 3 days before harvesting.

### Construction of SOX2-knock-in-GFP-24 × MS2 Cell line

Three sgRNAs targeting the SOX2 3′ UTR were designed using GenScript Benchling and synthesized. sgRNAs were co-transfected with Cas9-GFP protein into PEFs. Transfected cells were sorted by FACS after 48 h. PCR and Sanger sequencing of isolated genomic DNA were used to assess editing efficiency.

To knock-in the linker-EGFP-Puro-24 × MS2 fragment into the *SOX2* 3′UTR locus, a 3.1 kb donor fragment was amplified from pSLEP-24 × MS2 with 5′-biotin-labeled primers. In vitro-transcribed sgRNA was synthesized using the HiScribe T7 Kit (NEB,Cat.No. E2050S). RNP complexes (Cas9 + sgRNA) and donor fragments were electroporated into PK15 cells. After puromycin selection, positive clones were identified *via* nested PCR and Sanger sequencing. The sequences are shown in Table S1.

### Genomic DNA extraction and PCR

Genomic DNA was extracted using the Universal Genomic DNA Extraction Kit (Takara, Cat.No.9765). Targeted fragments (200–300 bp) were PCR-amplified using high-fidelity DNA polymerase and purified with a gel extraction kit (Takara, Cat.No.9762). PCR conditions were set as follows: initial denaturation at 98 °C for 5 min, followed by 35 cycles of 98 °C for 10 s, 50 to 60 °C for 5 s, and 68 °C for 3 s, with a final extension at 68 °C for 10 min. The targeted fragments were amplified by PCR using KOD high-fidelity DNA polymerase (Toyobo, Cat.No. KMM201), which enables high-sensitivity amplification even with low initial input. Prime sequences were shown in Table S2.

### Live-cell imaging

Live-cell imaging was performed on an Olympus FV3000(Olympus, Cat.No.TP0000527,Tokyo, Japan) confocal microscope at 5% CO_2_ and 37 °C. Cells were seeded into imaging dishes, transfected with labeled constructs using Lipofectamine 3000(Thermo, Cat. No.L3000001), and stained with Hoechst 33,342(Sigma, Cat,No.B2261-25MG). Images were analyzed using ImageJ software (National Institutes of Health) and GraphPad Prism 8(GraphPad Software).

### Cell treatments

Cells were transiently permeabilized with 1% Tween-20(Sigma, Cat.No.11332465001) in DPBS (Sigma, Cat.No.D8537-100Ml) for 10 min, then washed and returned to culture medium. For 1,6-hexanediol (Sigma,Cat.No.240117-50G) (1,6-HD) treatment, cells were incubated in 5% 1,6-HD(Sigma, Cat.No.240117-50G) for 60s, washed, and returned to normal medium. Real-time monitoring, observation and fluorescence image acquisition of live cells were performed using an Olympus FV3000 laser scanning confocal microscope (Olympus; Cat. No. TP0000527).

### Immunofluorescence

Cells were fixed in 4% paraformaldehyde (Beyotime, Cat.No.P0099-100ml) for 30 min, permeabilized with 1% Triton X-100(Sigma, Cat.No.X100-5Ml) for 1 h at 37 °C, and blocked in 1% BSA(Sigma, Cat.No.V900933-100G). Primary antibody incubation was performed overnight at 4 °C, followed by secondary antibody incubation at 37 °C for 1 h. Nuclei were stained with Hoechst 33,342(Sigma, Cat. No.B2261-25MG). Coverslips were mounted with anti-fade reagent and imaged using a Nikon 80i microscope. The primary antibody was against SOX2 (anti-rabbit, sigma, Cat, No.S9072),the secondary antibody: donkey anti-rabbit (Invitrogen,IgG-546, Cat, No. A10040). Antibody specificity was confirmed by manufacturer data.

### Fluorescence analysis

Fluorescence images were analyzed using Fiji/ImageJ (National Institutes of Health). For line-scan analysis, a line was drawn across the region of interest, and pixel intensity was plotted along it. Data were normalized to the maximum intensity and analyzed using GraphPad Prism 8(GraphPad Software).

### Signal-to-noise ratio calculation

$$\text{SNR}=\text{SNR}=\frac{P\;\text{signal}}{P\;\text{background}}=\frac{M}{\text{SD}}$$

where M is the mean signal and SD is the standard deviation of the background.

### Time-fold calculation

**Figure:**
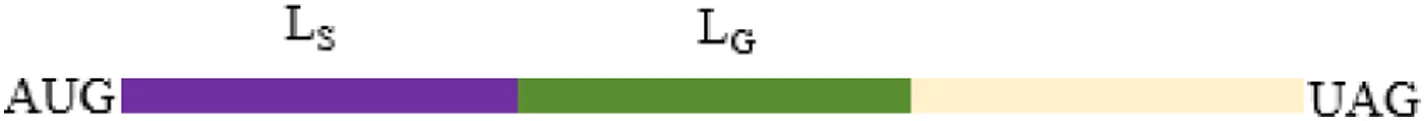

LS: SOX2 CDS sequence.

LG: inserted GFP sequence.

V0: elongation speed on regular sequence.

T = The time of translation fluctuations occur.

$$\text{Time}\;\text{Fold}=\frac{T-\frac{\mathrm{LG}}{\text{VG}}}{\frac{\text{LS}}{V0}}$$

### MATLAB analysis

Kinetic analysis of mRNA dynamics in live cells was performed using a custom MATLAB script based on previously published toolboxes (MATLAB R2018, MathWorks, Inc).

### Statistical analysis

All statistical analyses were performed using SPSS 16.0 (SPSS Inc.). Data are presented as mean ± standard error (SE) from at least three biological replicates. Statistical differences were analyzed using Student’s *t* test, with *p* < 0.05 considered statistically significant.

## Data availability

The data sets used and/or analyzed during the current study are available from the corresponding author on reasonable request.

## Supporting information

This article contains supporting information.

## Conflict of interest

The authors declare that they have no conflicts of interest with the contents of this article.
