# Supplementary material for: Dynamic SOX2 gene expression revealed by phase-separated single RNA imaging in live porcine cells
Source: J Biol Chem. 2026 Aug 11;302(10):113424. doi: 10.1016/j.jbc.2026.113424 (PMC13571416; doi:10.1016/j.jbc.2026.113424)
Supplement: Supplementary file 1 [file mmc1.docx]

Table S1. PCR primer sequences

| Primer ID | Sequence(5’->3’) |
| --- | --- |
| SOX2-5'HA-F | GCCCCCCCGTGGTTACCTCTTC |
| SOX2-5'HA-R | CATGTGAGAGAGAGGCAGTGTACC |
| SOX2-3'HA-F | GGGCCGGACAGTGAACTGGA |
| SOX2-3'HA-R | CT TGCCGGCATCGCGGTTTTTTG |
| KI-Identify primers-I-S | GGCTCAGTGGTCAAGTCCGA |
| KI-Identify primers-I-A | TTGAAGAAGTCGTGCTGCTTCATGT |
| KI-Identify primers-II-S | CGAGCTGGACGGCGACGTAAACGGCCACAAGTTCAGCGTGTCCGGCGAGGGCGAGGGCG |
| KI-Identify primers-II-A | AACACCGCCCCCGCTTCGACGCTCTCCGGCGTGGTCCAGACCGCCACCGCGGCGCCGTC |

Table S2. Sequences of Knockin-vector

| Sequence ID | Sequence(5’->3’) |
| --- | --- |
| Foldon-linker fragment: | GGTTATATCCCGGAAGCACCGCGTGATGGTCAGGCGTATGTTCGTAAAGATGGTGAATGGGTTCTGCTGAGCACCTTTC TGGGAGGGAAGGGAGGAGGAGGAGGAGGAGGTAGC |
| SOX2-sgRNA | GCCCCCTCCAGTTCACTGTC |
| SOX2-5'HA | GCCCCCCCGTGGTTACCTCTTCTTCCCACTCCAGGGCGCCCTGCCAGGCCGGGGACCTACGGGACATGATCAGCATGTACCTCCCCGGCGCTGAGGTGCCAGAGCCCGCCGCCCCCAGCAGACTTCACATGTCCCAGCACTACCAGAGCGGCCCGGTGCCCGGCACGGCCATCAACGGTACACTGCCTCTCTCTCACATG |
| SOX2-3'HA | GGGCCGGACAGTGAACTGGAGGGGGCGGGGGGAGAAAATTTTCAAAGAAAAAGAGGGAAATGGGAGGAGAGTAAGAAACAGTATGGAGAAAAACCCGGTACGCTTAAAAAAAAAAAAAATCATCACCCACAAAAAAAAATGACATCTGCAAAAGAGAACACCAATCCCATCCACACTCAAAAAACCGCGATGCCGGC |
